# A relative L‐arginine deficiency contributes to endothelial dysfunction across the stages of the menopausal transition

**DOI:** 10.14814/phy2.13409

**Published:** 2017-09-14

**Authors:** Jelena Klawitter, Kerry L. Hildreth, Uwe Christians, Wendy M. Kohrt, Kerrie L. Moreau

**Affiliations:** ^1^ Department of Anesthesiology University of Colorado Anschutz Medical Campus Aurora Colorado; ^2^ Division of Geriatric Medicine University of Colorado Anschutz Medical Campus Aurora Colorado; ^3^ Denver Veterans Administration Medical Center Geriatric Research Education and Clinical Center Denver Colorado

**Keywords:** Aging, endothelial function, estrogen, methylarginines, nitric oxide

## Abstract

Vascular endothelial function declines across the menopause transition in women. We tested the hypothesis that reduced availability of the endothelial nitric oxide synthase [eNOS] substrate L‐arginine is an underlying mechanism to vascular endothelial dysfunction across menopause stages. Endothelial function (brachial artery flow‐mediated dilation [FMD]) and plasma markers of L‐arginine metabolism (citrulline, N^G^‐mono‐methyl‐ւ‐arginine [L‐NMMA] asymmetric dimethylarginine [ADMA] and N^G^‐N^′G^‐dimethyl‐l‐arginine [SDMA]), were measured in 129 women: 36 premenopausal (33 ± 7 years), 16 early‐ (49 ± 3 years) or 21 late‐ (50 ± 4 years) perimenopausal, and 21 early‐ (55 ± 3 years) or 35 late‐ (61 ± 4 years) postmenopausal. FMD was progressively reduced across menopause stages (*P* < 0.001). Menopause stage was associated with L‐arginine concentrations (*P* = 0.012), with higher levels in early postmenopausal compared to early and late perimenopausal women (*P* < 0.05). The methylarginine and eNOS inhibitor L‐NMMA was higher in early and late postmenopausal women compared to premenopausal and early and late perimenopausal women (all *P* < 0.001), and was inversely correlated with FMD (*r* = −0.30, *P* = 0.001). The L‐arginine/L‐NMMA ratio, a potential biomarker of relative L‐arginine levels, was lower in postmenopausal compared to either premenopausal or perimenopausal women (both *P* < 0.001), and was positively correlated with FMD (*r* = 0.33, *P* < 0.001). There were no differences in plasma citrulline, ADMA or SDMA across groups. These data suggest that a relative L‐arginine deficiency may be a mechanism underlying the decline in endothelial function with the menopause transition in women. The relative L‐arginine deficiency may be related to elevated levels of the methylarginine L‐NMMA, which would compete with L‐arginine for eNOS and for intracellular transport, reducing NO biosynthesis.

## Introduction

One in three women dies from cardiovascular disease (CVD), making CVD a major public health problem in women (Go et al. 2014a,b). The menopause transition has been identified as a period of increased vascular vulnerability because the decline in ovarian function and sex hormone fluctuations are associated with alterations in CVD risk factors (e.g., abdominal fat distribution, impaired glucose tolerance, abnormal blood lipid profile, increased blood pressure) (Castelao and Gago‐Dominguez 2008; Schenck‐Gustafsson et al. 2011; Bassuk and Manson 2014). We recently demonstrated that vascular endothelial function becomes impaired during the perimenopausal period and worsens during the postmenopausal period, independent of age, CVD risk factors, physical activity, and aerobic capacity (Moreau et al. 2012a). Understanding the pathways involved in the development of endothelial dysfunction, a key trigger for atherosclerosis development (Moreau et al. 2012a), is important for preserving vascular health during this period of increased vascular risk in women.

Reduced availability of nitric oxide (NO), a vasoactive molecule that is critical for vascular health, is a triggering event in the pathogenesis of endothelial dysfunction and CVD (Duckles and Miller 2010; Knowlton 2012). Two key mechanisms underlying the reduction in NO bioavailability are reduced NO synthesis and increased NO inactivation by reactive oxygen species (Bernal‐Mizrachi et al. 2005; Gladwin and Kim‐Shapiro 2012). Endothelial NO is synthesized from its substrate L‐arginine, primarily by endothelial nitric oxide synthase (eNOS) and facilitated by the co‐factor tetrahydrobiopterin (BH_4_). eNOS stability plays a crucial role in regulating NO bioavailability and endothelial function. eNOS can become unstable and uncoupled if there are deficiencies in L‐arginine and/or any of its cofactors (e.g., BH_4_), resulting in an increased production of superoxide instead of NO and impaired endothelial function (Zhang et al. 2011). It is highly unlikely that systemic L‐arginine availability is a contributing factor to eNOS uncoupling because systemic arginine concentrations are 10‐30 times the levels needed intracellularly for NOS saturation. However, it is possible that alterations in L‐arginine metabolism create a relative L‐arginine deficiency (i.e., low levels of L‐arginine in the vicinity of eNOS despite normal plasma L‐arginine level) that could lead to eNOS uncoupling, reducing the bioavailability of NO (Bode‐Böger et al. 2007).

L‐arginine metabolism is complex and highly regulated. In addition to producing NO via eNOS, L‐arginine is metabolized by arginase to generate ornithine and urea. Because L‐arginine is a substrate for both eNOS and arginase, high arginase activity may limit L‐arginine availability for eNOS (Santhanam et al. 2008). L‐arginine can also be methylated to produce asymmetric dimethylarginine (ADMA), N^G^‐N^′G^‐dimethyl‐l‐arginine (SDMA) and N^G^‐mono‐methyl‐ւ‐arginine (L‐NMMA) (Bode‐Böger et al. 2007). ADMA and L‐NMMA can compete with L‐arginine for eNOS, whereas all three methylarginines can interfere with L‐arginine transport into endothelial cells, resulting in a relative L‐arginine deficiency (Scalera et al. 2004; Bode‐Böger et al. 2006; Bode‐Böger et al. 2007). Thus, increased metabolism of L‐arginine via the urea cycle and/or methylation could result in reduced L‐arginine bioavailability, eNOS uncoupling and reduced NO biosynthesis. In this study we examined whether our previously reported finding of impaired endothelial function in women across the stages of the menopause transition was associated with varying plasma levels of L‐arginine and other metabolites from the L‐arginine‐NO pathway.

## Methods

### Study population

Participant characteristics and brachial artery FMD have been previously reported (Moreau et al. 2012a). Briefly, participants were 129 healthy women aged 22–70 years. Premenopausal women (*N* = 36) had regular menstrual cycles with no change in observed cycle length (21–35 days), and perimenopausal (early peri *n* = 16; late peri *n* = 21) and postmenopausal (early post *n* = 21; late post *n* = 35) status were defined according to the Stages of Reproductive Aging Workshop (STRAW) criteria (Soules et al. 2001). Inclusion criteria were: (1) normotensive (resting blood pressure <140/90 mmHg), (2) fasted glucose <126 mg/dL, (3) nonsmoker, and (4) healthy as determined by medical history, physical examination, standard blood chemistries (chemistry panel, complete blood count, and thyroid stimulating hormone levels) and electrocardiography (ECG) at rest and during incremental treadmill exercise. Additionally, participants had not used oral contraceptives or hormone therapy (HT) for at least 6 months, were not taking medications that could influence cardiovascular function (i.e., antihypertensive, lipid lowering medications), and had not used vitamin supplements or anti‐inflammatory medications for at least 4 weeks prior to the vascular visit. All subjects gave their written informed consent to participate. All procedures were reviewed and approved by the Colorado Multiple Institutional Review Board (COMIRB).

### Measurements

Women were studied in the supine position following an overnight fast with proper hydration (water drinking only) and abstinence from caffeine. The participants' normal dietary patterns were maintained including sodium intake for the 2‐day period immediately prior to any measurements. Premenopausal and perimenopausal (when possible) women were tested 7–10 days after onset of menstruation (i.e., mid‐follicular phase) so that vascular comparisons between premenopausal and perimenopausal would be at a similar cycle phase. Late perimenopausal women were tested regardless of menstrual cycle phase if after 2 months the women did not have a cycle. The study took place at the University of Colorado Denver Colorado Clinical and Translational Sciences Institute (CCTSI) Clinical and Translational Research Center (CTRC).

#### Endothelial function

Ultrasound measurements of brachial artery flow‐mediated dilation (FMD) were performed as previously described in detail by our laboratory (Moreau et al. 2012a,b). Brachial artery diameter and blood flow velocity were acquired at baseline. Reactive hyperemia was produced by inflating the cuff to 250 mmHg of pressure for five minutes followed by rapid deflation. After the release of the arterial occlusion, Doppler blood flow velocity was acquired and B‐mode ultrasound brachial artery diameter images were measured continuously for two minutes. Brachial artery diameter and flow velocity were acquired and analyzed using a commercially available software package (Vascular Analysis Tools MIA, Coralville, IA) by the same individual. All participants achieved a peak diameter before the end of the two minutes post‐cuff deflation. All images were coded by number and blinded to group assignment. All procedures adhered to published guidelines for assessing FMD in humans (Corretti et al. 2002). The coefficient of variation and intra‐class correlation coefficient for trial‐to trial reliability measured in 10 individuals for baseline brachial artery diameter, peak diameter and FMD (%) were 2% and 0.97, 1.5% and 0.99, and 2.2% and 0.99, respectively.

#### Brachial artery blood pressure, body composition, metabolic risk factors, sex hormones, and dietary analysis

Peripheral arterial blood pressure was measured in triplicate in the seated and the supine positions with a semi‐automated device (Dinamap, Critikon/Johnson & Johnson, Tampa, FL) over the brachial artery, as previously described (Tanaka et al. 2000). Total (percent of total mass) and regional (percent of mass in trunk region) body fat were determined using dual energy x‐ray absorptiometry (Hologic Discovery W, Hologic Inc, Waltham, MA). Minimal waist and hip circumferences were measured according to published guidelines and waist to hip ratio (WHR) was calculated (Lohman et al. 1988).

Fasted plasma concentrations of glucose, insulin, total‐ (Roche Diagnostic Systems, Indianapolis, IN) and high‐density‐lipoprotein (HDL‐C, Diagnostic Chemicals, Oxford CT) cholesterol were determined using enzymatic/colorimetric methods, and low‐density‐lipoprotein cholesterol (LDL‐C) was determined using the Friedewald equation (Friedewald et al. 1972). Serum concentrations of follicle stimulating hormone (FSH), estradiol, progesterone and sex hormone binding globulin were measured using chemiluminescense, estrone using radioimmunoassay and total testosterone using 1‐step competitive immunoassay. All assays were performed by the CCTSI CTRC core laboratory.

In a subpopulation of women (*N* = 70), dietary composition including arginine, and caloric intake were determined from 3‐day food intake as described previously (Stevenson et al. 1995). The CCTSI CTRC Nutrition Core analysed the dietary food records.

#### Arginine, arginine metabolites, and methylarginines

Plasma levels of arginine and its byproducts (citrulline, and in a subgroup of premenopausal and postmenopausal women due to sample volume, ornithine) and the methylarginines L‐NMMA, ADMA, and SDMA, were quantified using a validated high‐performance liquid chromatography‐tandem mass spectrometry (HPLC‐MS/MS) assay as previously described (Klawitter et al. 2014). Briefly, the API5000 mass spectrometer (AB Sciex, Concord, ON) was run in the positive electrospray ionization mode (ESI) using multiple reaction monitoring (MRM). The following ion transitions were used: Arg: m/z (mass/charge)= 175.2→70.1; ADMA: m/z = 203.2→46.2; citrulline: m/z = 176.4→70.1, L‐NMMA: m/z = 189.3→116.2, ornithine: m/z = 133.1→70.3; SDMA: m/z = 203.2→172.2; d7‐ADMA (internal standard): m/z = 210.2 →77.2. After addition of the internal standard solution (50 *μ*L of 50 *μ*mol/L d7‐ADMA), protein was precipitated from 200 *μ*L of plasma with 400 *μ*L of 0.1% formic acid in acetonitrile solution. The sample was vortexed for 5 min, centrifuged for 10 min at 13,000 g and transferred into an HPLC vial. Twenty *μ*L of the supernatant was injected onto a 4.6 × 12.5 mm guard column (Eclipse XDB‐C8, 5 *μ*m, Agilent Technologies, Palo Alto, CA) in line with a 3.0 × 150 mm analytical column (RP‐Amide, 3.5 *μ*m, Supelco, St. Louis, MO). The HPLC gradient started at 3% methanol and 97% 10 mmol/L ammonium formate buffer. A flow of 0.8 mL/min was maintained throughout the assay. At minute 4.5, the solvent gradient reached 25% methanol; hereafter methanol and was gradually increased to 98% within the next 3 min; after this the gradient was at 98% methanol for an additional 1.5 min, and then the columns were re‐equilibrated to the starting conditions for the remaining 2 min of the assay. The L‐arginine/ADMA and L‐arginine/L‐NMMA ratios were calculated because it has been suggested that L–arginine levels expressed relative to its competitive antagonists may reflect NOS substrate availability better than L‐arginine alone (Luneburg et al. 2011).

### Statistical analysis

Results are presented as mean ± standard deviation (SD) (for normally distributed), or median and interquartile range for skewed descriptive variables (e.g., estradiol, estrone, triglycerides) presented in tables, and log transformed for skewed L‐arginine derivatives (i.e., L‐NMMA, and L‐arginine/L‐NMMA ratio) presented in figures. For statistical comparison, skewed variables were log transformed. ANOVA was used to assess group differences and *P* < 0.05 was considered significant. Significant results were further evaluated with Tukey *post hoc* comparisons to identify group differences. Exploratory analyses were performed using Pearson product‐moment correlations to test for the presence of significant linear bivariate relations between variables of interest. Partial correlations were used to control for the effects of age, and CVD risk factors. Data analysis was performed with SPSS software, version 21.0 (IBM/SPSS, Armonk, NY).

## Results

### Participant characteristics

The majority (71.2%) of the study participants were Caucasian. Twenty‐seven percent of early postmenopausal women were prior HT users with an average duration of 3.2 ± 2.4 years, whereas 73% of late postmenopausal women had used HT in the past for an average duration of 5.8 ± 4.6 year. Compared to premenopausal women, age, trunk fat, seated systolic blood pressure, total (TC) and LDL cholesterol, and FSH concentrations were higher, and maximal aerobic power, estradiol, estrone, and testosterone concentrations were lower across the stages of the menopause transition (Table 1, all *P* < 0.05). There were no differences in dietary caloric intake, macronutrients or arginine across the groups (all *P* > 0.24; Table 2).

**Table 1:** Clinical characteristics

Variable	Pre *N* = 36	Early Peri *N* = 16	Late Peri *N* = 21	Early Post *N* = 21	Late Post *N* = 35	*P*‐value
Age, years	33 ± 7	49 ± 3	50 ± 4	55 ± 3	61 ± 4	<0.001
Weight, kg	66.1 ± 13.6	67.2 ± 9.3	66.9 ± 13.3	73.9 ± 13.2	70.5 ± 13.8	0.21
BMI, kg/m^b^	24.1 ± 5.6	25.2 ± 3.0	24.1 ± 4.3	27.7 ± 5.0	26.5 ± 4.7	0.03
Trunk fat, %	29 ± 9	33 ± 7	34 ± 8	39 ± 6	39 ± 6	<0.001
Waist circumference, cm	78 ± 8	82 ± 9	82 ± 13	87 ± 14	85 ± 10	0.02
WHR	0.79 ± 0.06	0.80 ± 0.07	0.80 ± 0.06	0.81 ± 0.08	0.81 ± 0.05	0.59
SBP, mmHg	108 ± 8	115 ± 11	116 ± 13	118 ± 13	121 ± 13	<0.001
DBP, mmHg	69 ± 7	74 ± 7	72 ± 8	74 ± 8	73 ± 9	0.12
HR, bpm	65 ± 10	62 ± 8	64 ± 10	65 ± 6	64 ± 7	0.80
Total cholesterol, mg/dL	153 ± 30	164 ± 26	168 ± 33	185 ± 32	194 ± 30	<0.001
LDL cholesterol, mg/dL	88 ± 24	97 ± 29	100 ± 31	112 ± 30	120 ± 29	<0.001
HDL cholesterol, mg/dL	48 ± 11	50 ± 8	51 ± 8	49 ± 13	52 ± 11	0.68
Triglycerides, mg/dL^a^	80 (67–140)	79 (58–99)	83 (78–106)	80 (67–140)	97 (75–131)	0.007
Fasted glucose, mg/dL	84 ± 8	87 ± 7	81 ± 8	89 ± 12	86 ± 9	0.03
Fasted insulin, *μ*IU/mL^a^	6.0 (3.8–10.3)	4.0 (3.3–8.0)	4.0 (3.0–8.5)	7.0 (4.5–13.5)	6.0 (4.0–12.0)	0.27
FSH, *μ*IU/mL	5.4 ± 3.0	27.6 ± 35.3	67.8 ± 37.4	72.6 ± 27.4	83.0 ± 29.2	<0.001
Estradiol, pg/mL^a^	64.0 (40.5–92.0)	54.0 (31.0–139.0)	39.0 (10.0–118)	11.0 (10.0–15.5)	10.0 (10.0–13.5)	<0.001
Estrone, ng/dL^a^	46.5 (35.8–68.3)	61.5 (37.0–89.3)	42.0 (28.0–72.5)	29.0 (23.0–39.5)	25.0 (20.0–36.5)	<0.001
Testosterone, ng/dL^a^	30.0 (19.0–45.0)	26.5 (17.0–35.8)	20.0 (17.0–25.5)	19.0 (17.0–29.0)	17.0 (17.0–24.8)	0.003
VO_2_peak, mL/kg/min^b^	33.6 ± 7.1	28.1 ± 4.7	27.5 ± 5.4	24.5 ± 3.1	22.9 ± 3.8	<0.001
LTPA, MET‐hr/wk^c^	16.7 ± 11.8	17.4 ± 10.8	14.6 ± 11.8	15.3 ± 21.2	12.8 ± 13.8	0.86
Brachial FMD, %	9.8 ± 2.3	7.5 ± 2.3	6.5 ± 2.0	5.6 ± 1.9	4.8 ± 1.9	<0.001

Data are mean ± standard deviation unless otherwise stated. Pre, premenopausal; Peri, perimenopausal; Post, postmenopausal; BMI, body mass index; WHR, waist hip ratio; SBP, systolic blood pressure; DBP, diastolic blood pressure; HR, heart rate; LDL, low‐density lipoprotein; HDL, high‐density lipoprotein; FSH, follicle stimulating hormone; VO_2_ peak, peak aerobic power; LTPA, leisure time physical activity; FMD, flow‐mediated dilation.

a Data are median (interquartile range)

b Sample sizes of 116

c Sample sizes of 109.

**Table 2:** Dietary intake of energy, macronutrients, and arginine

Variable	Premenopausal	Early Perimenopausal	Late Perimenopausal	Early Postmenopausal	Late Postmenopausal
*n*	16	12	18	12	12
Energy (kcal)	1694 ± 385	2072 ± 641	1838 ± 470	1735 ± 299	1739 ± 464
Fat (g)	62 ± 22	76 ± 31	70 ± 23	60 ± 15	75 ± 22
Carbohydrate (g)	203 ± 55	255 ± 103	227 ± 80	222 ± 7	195 ± 77
Protein (g)	71 ± 17	87 ± 24	80 ± 26	70 ± 14	75 ± 22
Arginine (g)	3.7 ± 1.1	4.7 ± 1.5	4.3 ± 1.4	3.8 ± 0.9	4.1 ± 0.9

Data are mean ± SD.

### Brachial artery FMD, plasma arginine, and arginine metabolites

**Figure 1:**
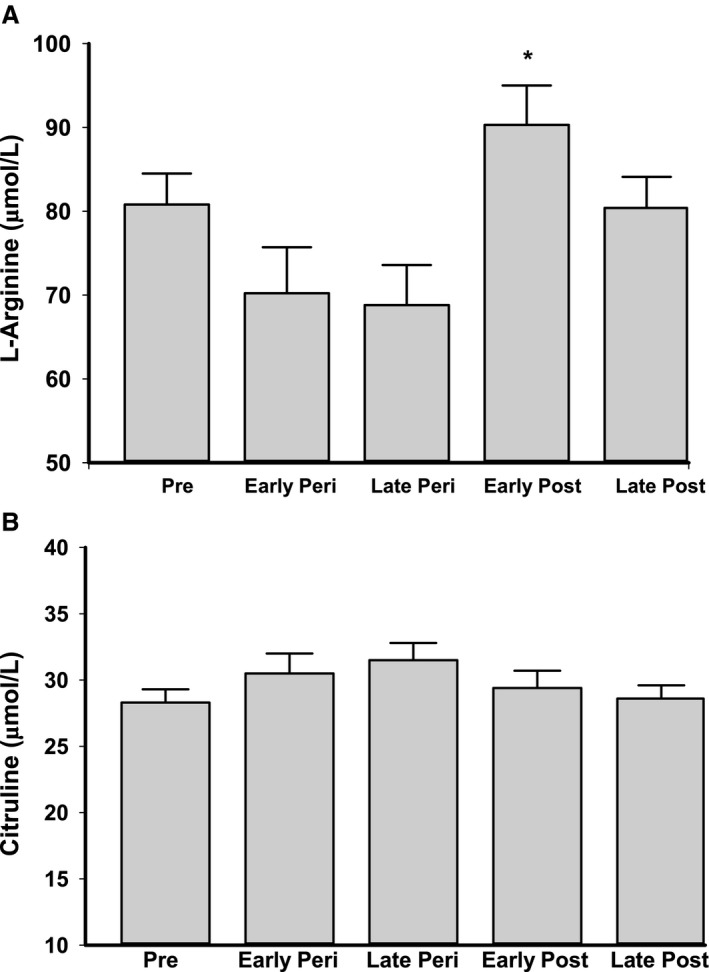
Plasma levels of (A) L‐arginine and (B) citrulline in premenopausal (pre), early and late perimenopausal (peri), and early and late postmenopausal (post) women. Data are presented as mean ± SE. Significance levels: *P < 0.05 versus early and late perimenopausal women.

**Figure 2:**
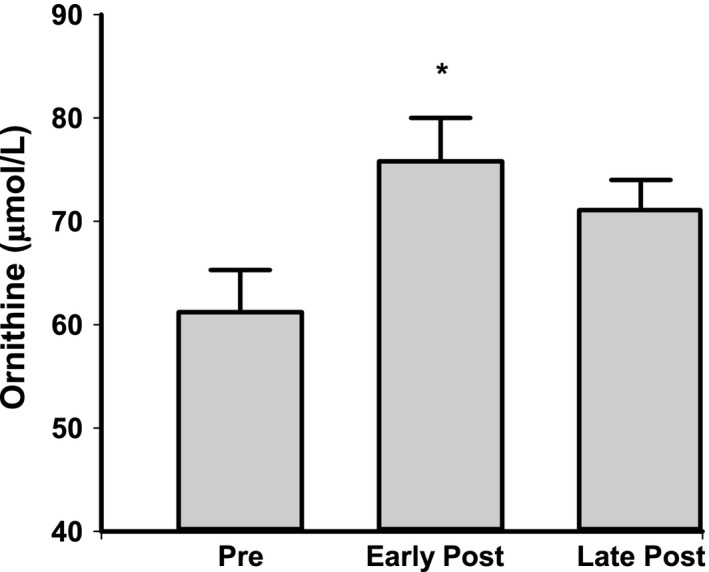
Plasma levels of ornithine in premenopausal (pre), and early and late postmenopausal (post) women. Data are presented as mean ± SE. Significance levels: *P < 0.05 versus premenopausal women.

Brachial artery FMD was progressively lower across the stages of the menopause transition (*P* < 0.001, Table 1): (1) compared to premenopausal women, FMD was lower in early and late peri‐and postmenopausal women (all *P* < 0.005), (2) compared to early perimenopausal, FMD was lower in postmenopausal, and (3) compared to late perimenopausal, FMD was lower in late postmenopausal women. There was a significant effect of menopause stage on L‐arginine concentrations (*P* = 0.012, Fig. 1A), with higher levels in early postmenopausal compared to early and late perimenopausal women (both *P* < 0.05). L‐arginine concentrations were not different between postmenopausal and premenopausal women, or perimenopausal and premenopausal women (all *P* > 0.27). There were no differences in citrulline levels among the groups (*P* = 0.32, Fig. 1B). In a subgroup of premenopausal and postmenopausal women, ornithine concentrations were higher in early postmenopausal (*n* = 14, *P* < 0.05, Fig. 2) and tended to be higher in late postmenopausal women (*n* = 29, *P* = 0.08) compared to premenopausal women (*n* = 15).

**Figure 3:**
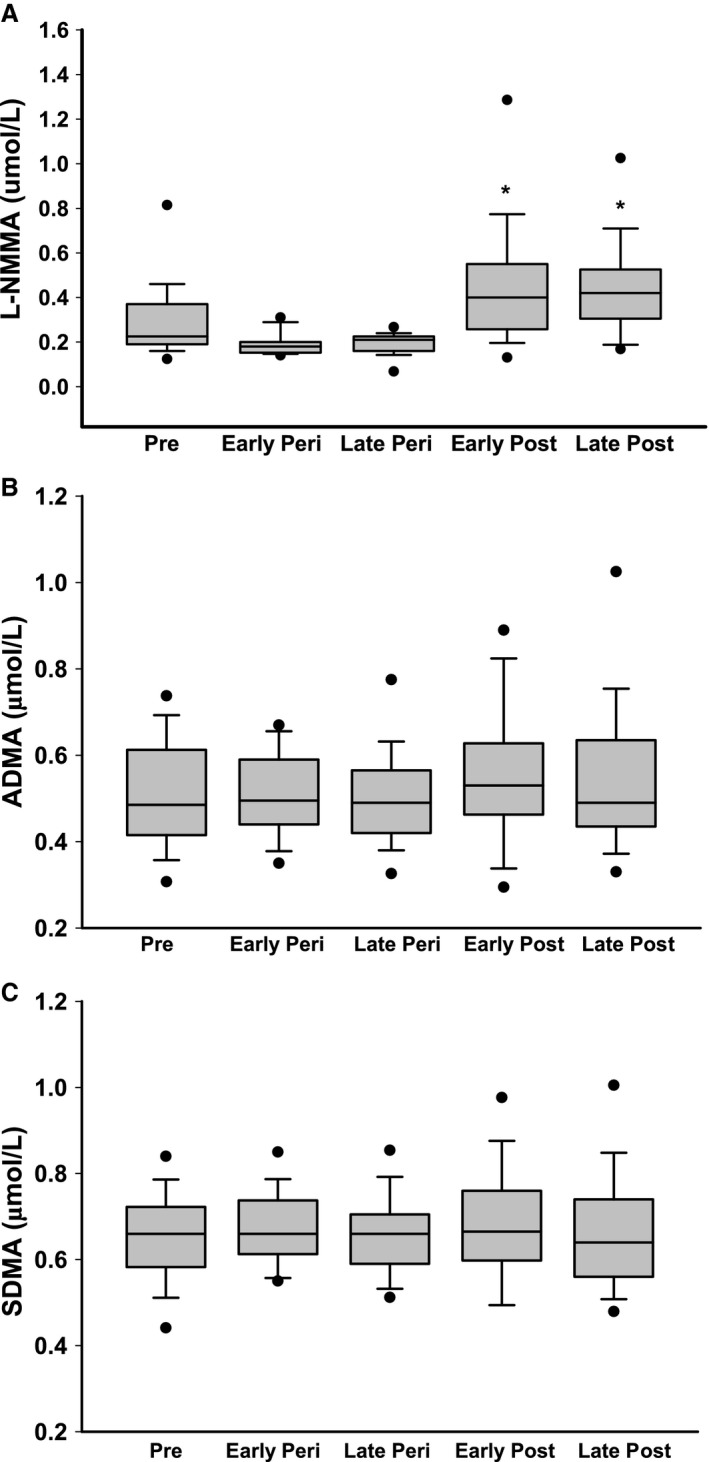
Plasma levels of (A) L‐NMMA, (B) ADMA and (C) SDMA in premenopausal (pre), early and late perimenopausal (peri), and early and late postmenopausal (post) women. Data are Median‐interquartile range and outliers are included at the 5th/95th percentile. Significance levels: *P < 0.0001 versus premenopausal and early and late perimenopausal women.

**Figure 4:**
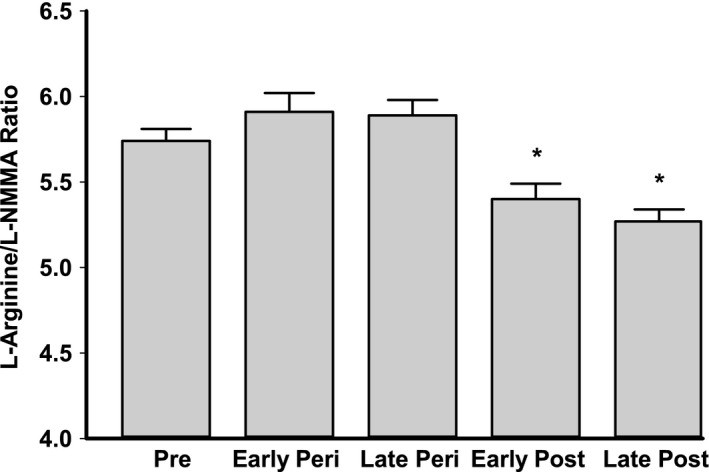
L‐arginine/L‐NMMA ratio, a measure of L‐arginine availability, in premenopausal (pre), early and late perimenopausal (peri), and early and late postmenopausal (post) women. Data are log transformed and are presented as mean ± SE. Significance levels: *P < 0.0001 versus premenopausal and early and late perimenopausal women.

The concentration of the methylarginine and eNOS inhibitor L‐NMMA was higher in early and late postmenopausal women compared to premenopausal and early and late perimenopausal women (Fig. 3A, *P* < 0.001). There were no differences in the levels of ADMA or SDMA among the groups (all *P* > 0.31, Fig. 3B and C). The L‐arginine/L‐NMMA ratio was lower in postmenopausal compared to premenopausal and perimenopausal women (*P* < 0.001, Fig. 4), whereas there tended to be an overall main effect of menopause stage (lowest levels observed in perimenopausal women) on the L‐arginine/ADMA ratio (*P* = 0.07, data not shown).

### Correlation analyses

**Figure 5:**
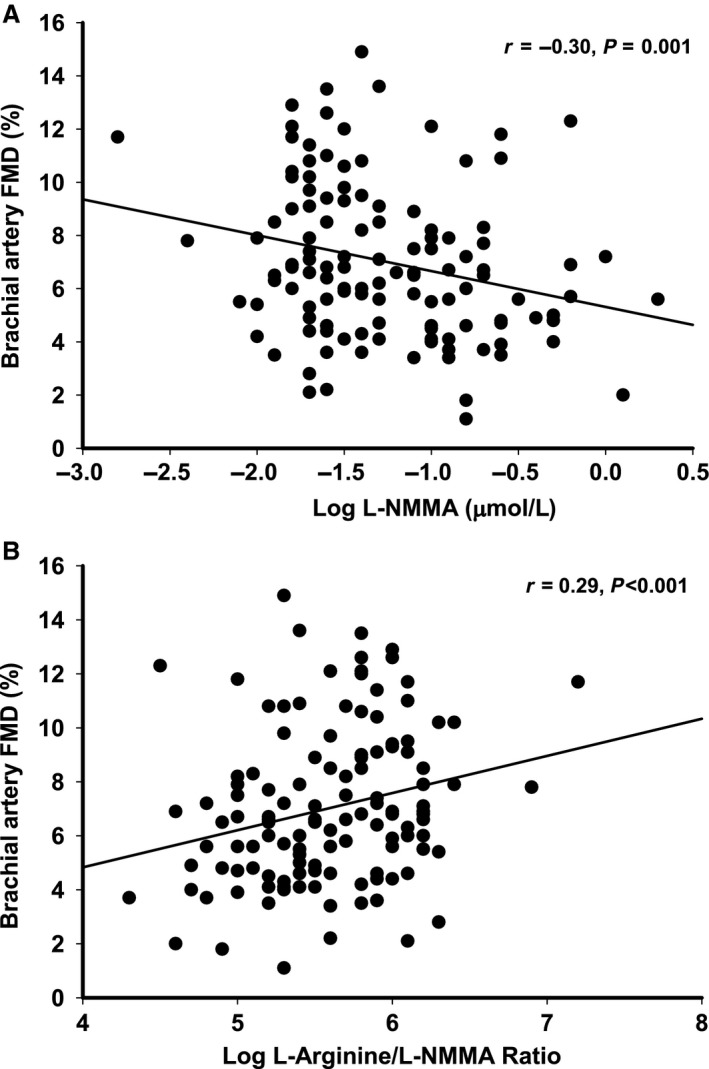
Brachial artery FMD was inversely related to (A) plasma L‐NMMA, and positively related to (B) L‐arginine/L‐NMMA ratio.

L‐NMMA was inversely correlated with FMD (*r* = −0.30, *P* = 0.001; Fig. 5A), estradiol (*r* = −0.44, *P* < 0.001) and estrone (*r* = −0.30, *P* = 0.001), and positively correlated with FSH (*r* = 0.24, *P* = 0.005) and age (*r* = 0.22, p‐0.01). Ornithine was also inversely correlated with estradiol (*r* = −0.36, *P* = 0.007). The L‐arginine/L‐NMMA ratio was positively correlated with FMD (*r* = 0.33, *P* < 0.001, Fig. 5B) and estradiol (*r* = 0.43, *P* < 0.001) and estrone (*r* = 0.27, *P* = 0.003) and inversely with FSH (*r* = −0.23, *P* = 0.008). Within the L‐arginine metabolic derivatives, L‐arginine correlated positively with L‐NMMA (*r* = 0.40, *P* < 0.001), ADMA (*r* = 0.56, *P* < 0.001), SDMA (*r* = 0.28, *P* = 0.001) and citrulline (*r* = 0.32, *P* < 0.001). L‐NMMA was correlated with ADMA (*r* = 0.33, *P* < 0.001), and ADMA was correlated with SDMA (*r* = 0.54, *P* < 0.001) and citrulline (*r* = 0.41, *P* < 0.001). L‐arginine/L‐NMMA and L‐NMMA were also correlated with age (*r* = −0.26, and *r* = 0.22, respectively both *P* < 0.05), total cholesterol (*r* = −0.19 and *r* = 0.18, respectively, *P* < 0.05) and LDL‐cholesterol (*r* = −0.19, and *r* = 0.20, respectively, *P* < 0.05). The correlation between FMD and L‐arginine/L‐NMMA (*r* = 0.22, *P* = 0.014) and FMD and L‐NMMA (*r* = −0.21, *P* = 0.018) remained after controlling for age, total and LDL cholesterol.

## Discussion

This study provided novel insight into the potential role of reduced L‐arginine bioavailability as a contributing factor to endothelial dysfunction across the stages of the menopause transition in healthy women. Specifically, we found that postmenopausal women had elevated levels of the metabolic by‐product ornithine compared to premenopausal women, which may be indicative of augmented L‐arginine metabolism via arginase. Additionally, compared to premenopausal and perimenopausal women, postmenopausal women had elevated levels of the methylarginine and eNOS inhibitor L‐NMMA, and a reduced L‐arginine/L‐NMMA ratio, a measure reflective of eNOS substrate availability. Moreover, L‐NMMA was inversely correlated and the L‐arginine/L‐NMMA ratio was positively correlated with brachial artery FMD and circulating estradiol concentrations.

### L‐arginine and endothelial function

We previously showed a progressive impairment in vascular endothelial function, measured via brachial artery FMD, across the stages of the menopause transition in healthy women, independent of age and adverse CVD risk factors (Moreau et al. 2012a). We speculated that the impairment in endothelial function was likely mediated by reductions in the endothelium‐derived vasodilating molecule NO. Brachial artery FMD implemented using a similar methodology as that employed in the present study (i.e., 5 min occlusion, distal cuff placement) is thought to be a “bioassay” of NO availability because it has been shown to be mediated predominantly (98%) by NO in young healthy men (Doshi et al. 2001), although the contribution of NO can vary based on age (~50% in older men), disease state and vessel size (Stoner et al. 2012). One mechanism for the loss of NO bioavailability is reduced synthesis of NO. Nitric oxide is produced from the semi‐essential amino acid L‐arginine catalyzed by eNOS, and thus, NO synthesis is dependent on both L‐arginine availability and eNOS function.

Decreased bioavailability of L‐arginine plays a role in a variety of diseases including those with cardiovascular complications (Madigan and Zuckerbraun 2013; Pernow and Jung 2013; Rajapakse and Mattson 2013; Tang et al. 2013). In the present study, an interesting U‐shaped pattern of L‐arginine concentrations was observed across menopause stages, with a progressive reduction in arginine level across premenopausal to perimenopausal women, then higher levels in postmenopausal women that were significantly higher than perimenopausal women but similar to premenopausal women. It is unclear why L‐arginine concentration appeared to “recover” in the postmenopausal women, but could represent a compensatory response to increase NO biosynthesis, as postulated previously by authors who observed similarly elevated L‐arginine in hypertensives with endothelial dysfunction compared to normotensives (Moss et al. 2004; Perticone et al. 2005, 2010). This compensatory signal to increase NO biosynthesis could be related to a relative L‐arginine deficiency within the vicinity of eNOS, that can occur when there is an imbalance between L‐arginine synthesis and L‐arginine metabolism, and/or inhibition of L‐arginine cellular transport (Moss et al. 2004; El‐Hattab et al. 2012).

### Relative L‐arginine deficiency and endothelial dysfunction: possible role of altered arginine synthesis

Arginine is derived from the diet, whole body protein turnover, and de novo synthesis from citrulline via the argininosuccinate synthase (ASS) and argininosuccinate lyase (ASL) pathway; the latter represents ~60% of de novo arginine synthesis, but only 5–15% of circulating arginine concentrations (Morris 2006). Citrulline can also be synthesized from arginine via arginase to ornithine and the ornthinine carbamoyltransferase (OTC) pathway (Morris 2006). Endothelial cells also contain the enzymes ASS and ASL, thus, NO can also be produced from recycled L‐arginine from citrulline. Reduced dietary arginine intake or impaired de novo synthesis of L‐arginine could therefore contribute to a relative L‐arginine deficiency and subsequent endothelial dysfunction. In this study, dietary arginine intake and citrulline concentrations were not different across the groups. However, it is important to note that dietary recall methods are not always accurate and systemic concentrations of citrulline do not necessarily reflect intracellular levels. Future investigations featuring labeled substrates should examine whether de novo synthesis of L‐arginine from citrulline is reduced at the local vascular level in women across menopause stages.

### Relative L‐arginine deficiency and endothelial dysfunction: possible role of arginase and altered arginine metabolism

One of the major catabolic enzymes that impacts the availability of L‐arginine for NO production is arginase, which exist in two isoforms: type I, located in the cytosol, and type II, located in the mitochondria (Morris 2005; Pernow and Jung 2013). Both isoforms convert L‐arginine into urea and ornithine, and both are expressed in endothelial cells. Although eNOS has a higher affinity for L‐arginine than arginases, arginases have a much higher Vmax than eNOS, and compete equally for L‐arginine (Pernow and Jung 2013). Thus, increased arginase activity could compete with eNOS for L‐arginine, causing a relative L‐arginine deficiency and resultant endothelial dysfunction across the menopause transition (Pernow and Jung 2013). In this regard, arginase activity and expression were shown to be elevated in ovariectomized rats fed a high cholesterol diet compared to sham and ovariectomized rats administered estradiol (Hayashi et al. 2006). Moreover, arginase inhibition improved endothelial function in older animals, and in patients with Type 2 diabetes and coronary artery disease (Kim et al. 2009; Shemyakin et al. 2012; Kövamees et al. 2016).

In the present study ornithine concentration was elevated in postmenopausal compared to premenopausal women, suggesting that arginase activity may be increased in postmenopausal women. Alternatively, the elevated levels of ornithine in postmenopausal women may not be related to greater de novo synthesis by arginase, but rather due to greater synthesis and/or metabolism of ornithine by OTC (Moretto et al. 2017). We recognize that because we did not measure ornithine levels in perimenopausal women or arginase levels across the groups, we are missing important information on L‐arginine metabolism via arginase in these women. Future investigations should examine this potential mechanism, along with the role of arginase in the decline in endothelial function with the menopause transition in women.

### Relative L‐arginine deficiency and endothelial dysfunction: possible role of methylarginines and altered arginine metabolism

In addition to the metabolism of L‐arginine by arginase, L‐arginine can be methylated by protein arginine methyltransferase (PRMT) to produce the methylarginines L‐NMMA, ADMA and SDMA. L‐NMMA and ADMA are potent inhibitors of NO production as they compete with L‐arginine for eNOS active sites (Vallance et al. 1992b; MacAllister et al. 1994; Leiper and Vallance 1999); SDMA does not appear to directly affect eNOS activity (Vallance et al. 1992a). At physiological extracellular levels of L‐arginine and in the presence of normal ADMA and L‐NMMA levels, eNOS is saturated with L‐arginine, resulting in NO production and vasodilation (Bode‐Böger et al. 2007). However, in the presence of physiological L‐arginine levels and pathophysiological ADMA and L‐NMMA levels, eNOS activity decreases and becomes uncoupled, leading to less NO production and impaired vasodilation (Bode‐Böger et al. 2007). Because circulating L‐NMMA concentrations are much lower than ADMA concentrations, the majority of studies have evaluated the link between elevated ADMA levels, endothelial dysfunction and the development of cardiovascular disorders (Vallance et al. 1992a; Perticone et al. 2005; De Gennaro et al. 2007; Juonala et al. 2007). Indeed, elevated ADMA levels have been thought to explain the “L‐arginine paradox,” the observation that acute and chronic L‐arginine supplementation improves endothelial function even though basal levels of L‐arginine in healthy and various patient populations are 15–35 times higher than the Michaelis‐Menten constant (K_M_) of eNOS for L‐arginine (Bredt and Snyder 1990; Bode‐Böger et al. 2003, 2007). In the setting of elevated competitive inhibitors (i.e., ADMA and L‐NMMA), exogenous L‐arginine supplementation displaces the competitive inhibitors and restores physiological L‐arginine/ADMA and/or L‐arginine/L‐NMMA ratio to a level that recouples eNOS activity (Bode‐Böger et al. 2007). In fact, intra‐arterial infusion of L‐arginine improved acetylcholine‐stimulated endothelial‐dependent vasodilation in adults with essential hypertension who had elevated L‐arginine concentrations compared to normotensives (Perticone et al. 2005). The authors speculated that the elevated L‐arginine levels were a counter‐regulatory response to compensate NO inhibition by ADMA, which was elevated in the hypertensive adults. L‐arginine and ADMA were positively correlated with each other, and inversely correlated with endothelial function, supporting the idea of a relative L‐arginine deficiency due to altered L‐arginine metabolism (Perticone et al. 2005). Consistent with this, in the present study L‐arginine levels were positively correlated with ADMA and L‐NMMA. In contrast to the previous observation (Perticone et al. 2005), we found no difference in ADMA concentrations across menopause stages; however, we did observe elevated L‐NMMA levels in postmenopausal compared to premenopausal and perimenopausal women. Moreover, the ratio of L‐arginine/L‐NMMA, which may reflect eNOS substrate availability better than L‐arginine levels alone, was significantly reduced in postmenopausal women, supporting the idea of a relative L‐arginine deficiency in the postmenopausal women. Finally, and importantly, L‐NMMA was inversely and the L‐arginine/L‐NMMA ratio positively correlated with brachial artery FMD, suggesting that a relative L‐arginine deficiency related to elevated levels of the eNOS inhibitor L‐NMMA, may be a contributing factor to the endothelial dysfunction in postmenopausal women.

To the best of our knowledge, this was the first study to demonstrate an elevated L‐NMMA level in postmenopausal women. Although, the absolute difference in circulating L‐NMMA between postmenopausal women and the other groups is small, intracellular L‐NMMA levels may exceed extracellular concentrations by ~5 fold (Leiper and Vallance 1999). The reasons for the elevation in L‐NMMA and not ADMA are not clear but could be related to reduced activity of the enzyme that catalyzes the metabolism of L‐NMMA (and ADMA), dimethylarginine dimethylaminohydrolase (DDAH), due to the loss of estrogen. Estradiol has been shown to stimulate DDAH activity and the metabolism of ADMA in human and murine endothelial cell lines (Holden et al. 2003), and in the present study higher estradiol concentrations were strongly correlated with lower L‐NMMA levels and a greater L‐arginine/L‐NMMA ratio. To the best of our knowledge no one has looked at estradiol in relation to L‐NMMA levels. It is plausible that DDAH hydrolyzed more ADMA than L‐NMMA, thus, explaining for the higher L‐NMMA levels and not ADMA levels in the postmenopausal women.

### Relative L‐arginine deficiency and endothelial dysfunction: possible role of reduced L‐arginine transport

The transport of extracellular L‐arginine into endothelial cells is essential for NO production (Moss et al. 2004; Rajapakse and Mattson 2009, 2013). The major transport systems responsible for uptake of extracellular L‐arginine into endothelial cells include plasma membrane y^+^L amino acid transporters and y+ cationic amino acid transporters. After transport into the endothelial cell L‐arginine is then coupled to eNOS and converted to NO and citrulline. Estradiol has been shown to augment L‐arginine transport in human umbilical venous endothelial cells (Bentur et al. 2015), and thus, the decline in estradiol with the menopause transition could reduce L‐arginine transport and intracellular L‐arginine levels in postmenopausal women. Additionally, because all three methylarginines and ornithine enter endothelial cells through the y+ transporter, the elevated L‐NMMA and ornithine levels in the postmenopausal women could result in a relative L‐arginine deficiency and reduced NO production due to competition with L‐arginine for cellular transport (Chen et al. 2013).

### Other potential mechanisms for a relative L‐arginine deficiency and endothelial dysfunction

**Figure 6:**
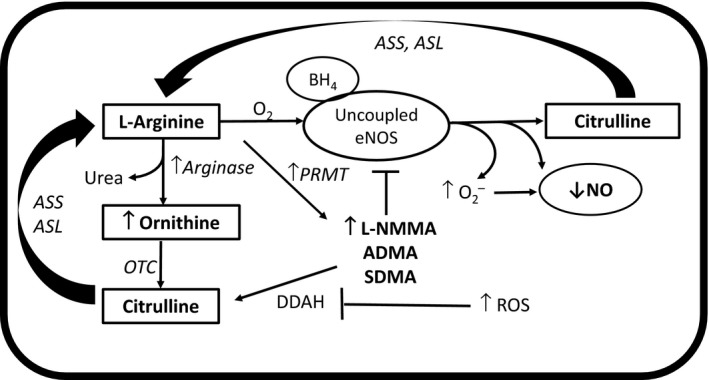
Scheme depicting how altered L‐arginine metabolism may contribute to endothelial dysfunction in postmenopausal women. In postmenopausal women, a relative L‐arginine deficiency may occur because of a greater metabolism of L‐arginine to ornithine due to increased arginase, and/or increased L‐arginine methylation to *N*^G^‐monomethyl‐l‐arginine (L‐NMMA). The elevated L‐NMMA would compete with L‐arginine for endothelial nitric oxide synthase (eNOS) binding sites. Elevated ornithine and L‐NMMA would also compete with L‐arginine for intracellular transport. A relative L‐arginine deficiency would uncouple eNOS, resulting in elevated superoxide (O_2_^−^) production and reduced NO. The O_2_^−^ would react with nitric oxide (NO), further reducing NO bioavailablity. Superoxide and other reactive oxygen species (ROS) could impair the activity of dimethylarginine dimethylaminohydrolase (DDAH) and increase L‐NMMA. BH_4_, tetrahydrobiopterin; O_2_, oxygen; ASS=argininosuccinate synthase; ASL, argininosuccinate lyase; PRMT, protein arginine methyltransferase; OTC, ornithine carbamoyltransferase.

We recognize that other mechanisms could contribute to a relative L‐arginine deficiency and endothelial dysfunction in postmenopausal women. In Figure 6 a schematic summary of our findings depict how altered L‐arginine metabolism may contribute to endothelial dysfunction in postmenopausal women. For example, when intracellular concentrations of L‐arginine are low (i.e., relative L‐arginine deficiency), eNOS exhibits a new catalytic function and uncouples, generating superoxide instead of NO (Druhan et al. 2008). Environments of reduced L‐arginine and/or BH_4_, L‐NMMA and ADMA have been shown to dose‐dependently increase superoxide production from uncoupled eNOS (Druhan et al. 2008). The increased superoxide would scavenge NO and decrease its bioavailability, causing an impairment in endothelial function. Moreover, elevated reactive oxygen species can impair the activity of DDAH resulting in elevated methylarginines, creating a vicious cycle of oxidative stress and endothelial damage. In this regard, estradiol was shown to reverse the effects of oxidized LDL on DDAH and ADMA (Monsalve et al. 2007), and we have previously demonstrated that oxidative stress contributes to endothelial dysfunction in postmenopausal women (Moreau et al. 2013). Future investigations should determine the contribution of a relative L‐arginine deficiency and/or elevated methylarginines (i.e., L‐NMMA) to oxidative stress‐mediated endothelial dysfunction in postmenopausal women.

### Limitations and experimental considerations

We used a cross‐sectional study design of healthy, non‐smoking sedentary women without clinical disease, and thus, we cannot generalize our findings to men or other patient populations, nor can our findings establish cause and effect. Additionally, we recognize that plasma concentrations of L‐arginine and its metabolic by‐products do not necessarily reflect levels seen at the local vascular level, and that L‐arginine metabolism is highly compartmentalized within cells. Future studies should examine how the loss of ovarian hormones impacts the synthesis, metabolism and transport of L‐arginine at the local vascular level.

We also recognize that we have not discussed the adverse effects of changes in CVD risk factors (i.e., adiposity, blood pressure, blood lipids, and lipoproteins) with the menopause transition on endothelial function. We previously reported that adjusting for elevated levels in adiposity, systolic blood pressure and total and LDL cholesterol did not alter the association between menopause stage and endothelial dysfunction (Moreau et al. 2012a). In the present study, we found that age and CVD risk factors were weakly to modestly correlated with L‐NMMA and L‐arginine/L‐NMMA ratio. Importantly, after adjusting for these factors both L‐NMMA and L‐arginine/L‐NMMA ratio were still significantly correlated with brachial artery FMD.

## Conclusion

In conclusion, our findings suggest that a relative L‐arginine deficiency and reduced NO biosynthesis may contribute to the impairment in endothelial function across the stages of the menopause transition, particularly in postmenopausal women, possibly related to the loss of estradiol. The relative L‐arginine deficiency may be related to elevated levels of the eNOS inhibitor L‐NMMA, increased metabolism of L‐arginine to ornithine via the urea cycle, possibly due to elevated arginase, and/or reduced L‐arginine transport into the cell. This relative L‐arginine deficiency could lead to an uncoupling of eNOS, resulting in greater superoxide production and less NO, impairing endothelial function. Future investigations should determine whether targeting this relative L‐arginine deficiency with either L‐arginine and/or citrulline supplementation over the course of the menopause transition could prevent the decline in endothelial function in women.

## Conflict of Interest

None.
